# Three-Dimensional Immersion Scanning Technique: A Scalable Low-Cost Solution for 3D Scanning Using Water-Based Fluid

**DOI:** 10.3390/s23063214

**Published:** 2023-03-17

**Authors:** Ricardo Spyrides Boabaid Pimentel Gonçalves, Jens Haueisen

**Affiliations:** Institut für Biomedizinische Technik und Informatik, Technische Universität Ilmenau, 98693 Ilmenau, Germany; jens.haueisen@tu-ilmenau.de

**Keywords:** 3D immersion scanning, radon transform, water 3D scanning, water level sensing, scalable 3D scanner, low-cost 3D scanning, liquid level sensing, CT scanner, 3D scanner

## Abstract

Three-dimensional scanning technology has been traditionally used in the medical and engineering industries, but these scanners can be expensive or limited in their capabilities. This research aimed to develop low-cost 3D scanning using rotation and immersion in a water-based fluid. This technique uses a reconstruction approach similar to CT scanners but with significantly less instrumentation and cost than traditional CT scanners or other optical scanning techniques. The setup consisted of a container filled with a mixture of water and Xanthan gum. The object to be scanned was submerged at various rotation angles. A stepper motor slide with a needle was used to measure the fluid level increment as the object being scanned was submerged into the container. The results showed that the 3D scanning using immersion in a water-based fluid was feasible and could be adapted to a wide range of object sizes. The technique produced reconstructed images of objects with gaps or irregularly shaped openings in a low-cost fashion. A 3D printed model with a width of 30.7200 ± 0.2388 mm and height of 31.6800 ± 0.3445 mm was compared to its scan to evaluate the precision of the technique. Its width/height ratio (0.9697 ± 0.0084) overlaps the margin of error of the width/height ratio of the reconstructed image (0.9649 ± 0.0191), showing statistical similarities. The signal-to-noise ratio was calculated at around 6 dB. Suggestions for future work are made to improve the parameters of this promising, low-cost technique.

## 1. Introduction

Computed tomography scanners (CT scanners) are employed in various settings, from industrial measurements to biomedical applications. However, the high cost and complexity of the equipment may limit their application [1,2].

Additionally, CT scanners can only acquire images of objects that fit inside their toroidal scanning area, so larger objects require larger and more expensive CT scanners [3].

The technology behind a CT scanner involves emitting an array of X-ray beams through an object to be scanned while measuring the attenuation of the X-rays on the other side [4,5]. A matrix with the attenuation values acquired during the scan is the equivalent of a radon transform of a slice of that object at that particular angle [3,6]. The CT scanner then rotates the beams around the object, repeating the process several times to generate radon transforms at different angles [5]. After turning at least 180${}^{\circ }$, these radon transforms are used to reconstruct a slice image of the original object [3,6].

The main difference between a tomograph and a 3D scanner is that the former can look inside the object being scanned, while the latter can only map its shape at a significantly lower cost [1,4].

There are several types of 3D scanners [3,7]. The most common are optical scanners, which use beams of light shining on the surface of an object and a camera analyzing the distortion of that light to generate a depth map and reconstruct the object [5,8].

However, reflective, transparent, and dark objects can be problematic to this technology because they distort the light rays or do not provide enough reflected light for the camera to analyze [9,10,11,12]. Additionally, light only travels in a straight line, so parts of the object containing gaps or non-straight holes may create shadows in the projected light, preventing the object from being adequately scanned [8].

The 3D Immersion Scanning Technique aims to address some of the limitations of CT scanners, such as their high cost, complexity, and scalability issues, as well as those of the optical scanning techniques that prevent the accurate capture of transparent, dark, or reflective objects.

Three-Dimensional Immersion Scanning involves immersing the object in a water-based fluid and reconstructing the image of the object by measuring the fluid level displacement during submersion with a needle tapping the surface of the fluid. The reconstruction approach is similar to that of CT scanners but without the need for dangerous X-rays. In addition, as light is also not used for scanning, transparent, reflective, dark color, and even gaps or irregularly shaped openings objects can be easily scanned.

Our technique may be a more accessible approach to industries and applications that may not have the resources to invest in expensive 3D or CT scanners. In addition, the adaptability to scan various object sizes and characteristics that pose a challenge to other scanning technologies makes this a very versatile scanning technology. Therefore, the proposed 3D Immersion Scanning Technique can significantly contribute to the field of 3D scanning and imaging.

## 2. Materials and Methods

### 2.1. Methods Overview

Assume that a cylinder is lying horizontally inside a CT scanner, but the X-ray beams that usually move in a circular trajectory around the object are now stationary, arranged in a vertical line on the side of the object, while the object is the one that rotates. By changing the static reference frame, we did not change the movement of the beam with respect to the object, so the technique will work as usual.

Each of the X-rays is activated sequentially, from the bottom to the top of the vertical array, generating a radon transform of that sliced area of the object. After that, the object rotates a bit, and the process repeats.

This is a good analogy to the working principles of this 3D Immersion Scanning Technique. In our technique, the cylinder is lowered stepwise inside the water, and the rising water level is precisely measured, similar to the sequential vertical activation of the X-ray beams. The larger the volume of the submerged object, the larger the increment in the water level, just like more extensive paths crossed by X-rays usually provide higher attenuation measurements.

After submerging the whole cylinder, the cylinder is brought back above the water and slowly spun to allow the water to drain. This prevents water from the previous submersion from causing perturbation in the surface level height when the object is submerged again. Then, the object is turned a fraction of the 180${}^{\circ }$ necessary to reconstruct the image, and the whole process is repeated. Ultimately, the data are processed in software similar to that of the CT scanner.

Despite the similarities between both techniques, the beam of X-rays in a CT scanner acquires information from a ring across the cylinder, while by submerging the cylinder in our technique, the whole submerged volume is responsible for the water level increment instead. Therefore, if the object to be scanned is not symmetrical along its axis of rotation, several different sliced images of the object will be overlapped in the final reconstructed image. Each of these parts will be detailed in the following sections.

### 2.2. The Platform’s Mechanical Setup

The setup to perform a 3D Immersion Scanning consists of a tank with water, a vertical lifting axis with a horizontal platform to hold the object to be scanned (that can be rotated in the horizontal axis up to 180${}^{\circ }$), and a water level sensor. The tank should be rigid enough to withstand rising water levels due to the object’s submergence without causing the walls to bend, introducing measurement errors. Thus, a rigid rectangular polypropylene reservoir from a local hardware store was used.

**Figure 1:**
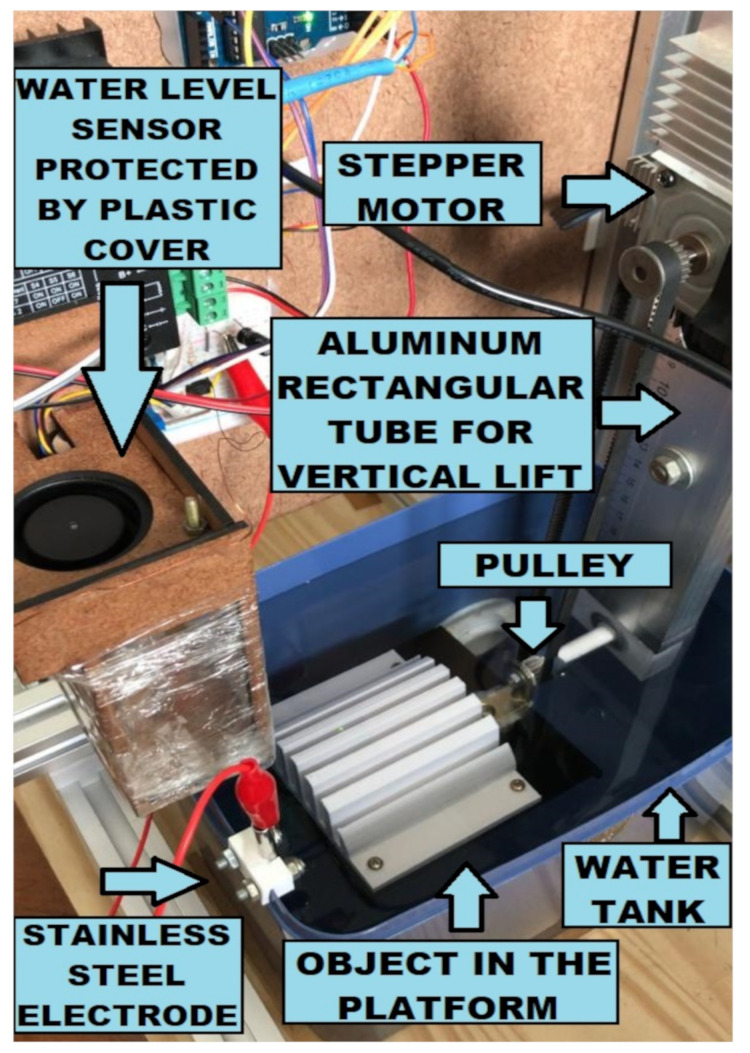
The mechanical setup of the 3D immersion scanner. The platform contains a white 3D printed model symmetric along its rotation axis. A pulley and a belt turn the platform with the object, while the aluminum tube lowers or lifts the platform.

Since the volume of water that rises is equal to the volume of the object being submerged, the increase in water level is proportional to the ratio between the volume of the submerged part of the object and the horizontal area of the tank. Therefore, the horizontal area of the tank should not be significantly larger than the area required to fit the object and the platform. This results in a more significant increase in water level with higher signal-to-noise ratio (SNR) measurements. A tank with a horizontal area of 21 × 18 cm was used to scan objects inside the 10 × 10 cm platform (Figure 1, blue). The depth of the tank should be large enough to submerge the object completely.

The platform that can be lifted and submerged by a vertical linear table (SLW-BB-16120, Igus, East Providence, USA), with a stepper motor (NEMA 23, Minebea, Bangkok, Thailand), is connected to a 26 cm long rectangular aluminum tube. On the top of the tube, another stepper motor (NEMA 17, Minebea, Bangkok, Thailand) connects the platform pulley through a belt so that water-sensitive parts stay dry while only the platform and the object submerge. Each stepper motor is controlled by micro-step drivers (TB6600, Toshiba, Tokio, Japan) connected to an Arduino microcontroller board (Mega 2560, Arduino, Ivrea, Italy).

### 2.3. Water Level Sensor

The water level sensor consists of a stainless-steel acupuncture needle connected to the non-inverting input of an LM339 comparator (ST Microelectronics, Geneva, Switzerland). The needle is connected to the GND pin (0 V) of the Arduino through a 4.7 MΩ pull-down resistor. Underwater, a stainless-steel rod is used as an electrode connected to the supply (5 V). The needle is moved vertically, back and forth through a micro linear slide (Hobby World, Jiangmen, China) close to the water. When the needle touches the water, the comparator voltage becomes high, indicating that the needle is currently at the water’s surface.

The threshold voltage at the inverting input of the LM339 is set so that the tip of the needle touching the water is enough to trigger the comparator.

The 4.7 MΩ resistor prevents an open-circuit configuration at the comparator when the needle is in the air and limits the electric current that flows between the needle and the underwater rod, reducing the oxidation and preventing electrolysis bubbles formation that could interrupt the circuit. The impedance formed by the water bridge between the needle-rod pair consists of a few Mega Ohms. Thus, the 4.7 MΩ resistor also provides a considerable voltage to the comparator, with high SNR. The comparator then transforms this voltage into a TTL logic signal, connected to a digital input of the Arduino.

**Figure 2:**
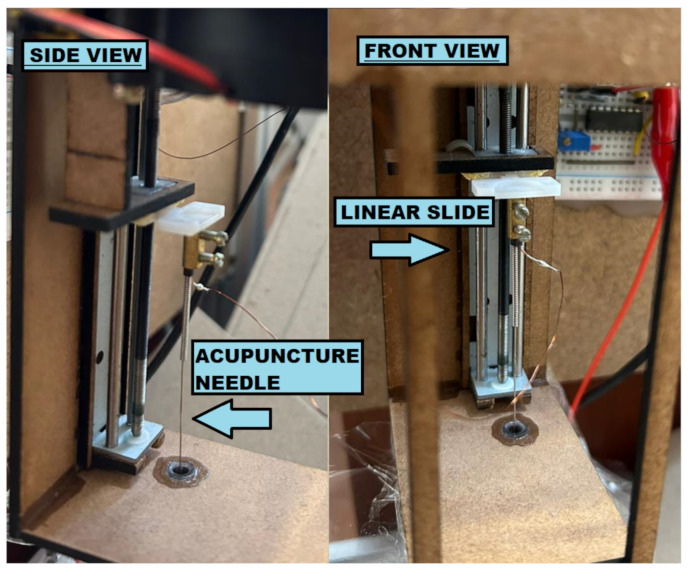
Water level sensor without the plastic protection cover: side and front views. The linear slide controls an acupuncture needle connected to a thin copper wire to detect the fluid level height.

The micro linear slide that moves the needle consists of a 1.5 mm × 10.5 cm linear slide with a 3 mm in diameter screw that is connected to a stepper motor, as in Figure 2. A precision of 25 µm/step in an 8 cm effective stroke can be obtained, and by also using the micro-step driver TB6600 to its stepper motor, the vertical resolution can be increased even more, if needed.

### 2.4. Immersion Scanning Procedure

Initially, the scanner lifts the platform until the infrared beam of the U-shaped limit sensor switch is interrupted. The system then sets this point as the home position of the track, from which stepper motor steps can begin to be counted. The same is performed with the water level sensor with its needle. The object to be scanned is fixed to the platform. Then, the platform submerges 1/100 of the total track, setting the first layer height. The water level sensor starts moving the needle towards the water while counting the steps of its stepper motor. When the comparator voltage switches on, the number of steps counted represents the water level height. Once five water level measurements are performed, the platform is submerged again, and the process is repeated for the rest of the 99 layers.

After 100 layers scan, the platform is raised all the way up. Now, outside water, the platform slowly rotates to drain any liquid from the object’s surface for about 3 s. This step is particularly important when using viscous fluids so that little fluid remains on the object during the next scan preventing artifacts.

The platform then rotates back to its original position, incrementing 3.6${}^{\circ }$, and the whole process repeats for a total of 50 times, where the platform will be at an angle of 180${}^{\circ }$ from the first scan, and there are enough acquisitions for an image to be computed.

### 2.5. Vibration Mechanisms

When the platform with the object is submerged in the water tank, vibrations appear on the surface of the liquid. These vibrations are caused not only by convection currents flowing in the liquid due to the submergence of the platform but also by systematic vibrations caused by the stepper motors.

In addition, every time the height of the water is measured, there is the need to subtract the height of the previously added layers to determine the height of a single layer of water. This is similar to differentiating the signal, which tends to amplify high-frequency noises.

Before measuring the water level, one could wait for the vibrations to decay exponentially over time. However, waiting a few seconds at each layer, for each angle of the platform, will cause an enormous amount of extra time to accumulate at the end of a scan.

Vibrations are non-stationary, depending on which part and shape of a particular object is being submerged, providing different noise profiles that are difficult to filter. Moreover, low-frequency filters in software would come at the expense of blurring the contours of the reconstructed image.

Therefore, we added 2.5 g/L of the thickener agent Xanthan gum to the water to reduce the vibrations. The system can now damp transitory vibrations much more quickly, improving the quality of the reconstructed image. As the liquid is more viscous, rather than measuring the constant height of the water, the water surface is probed while the fluid is still flowing over the object immediately after lowering the platform.

**Figure 3:**
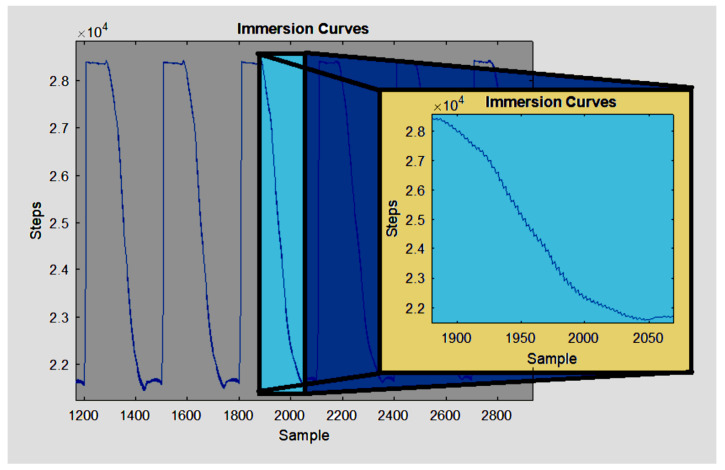
Curves measured by the water level sensor during submersion showing tiny damped step functions to be used as the AC signal in the lock-in amplifier.

The five measurements behave as five samples in a damped step function taken for each layer (Figure 3), and a set of damped step functions provide an AC signal that can be very precisely filtered through a lock-in amplifier implemented in MATLAB R2017b (Mathworks, USA), similar to [13].

### 2.6. Image Reconstruction

Finding the synchronous detection frequency of the lock-in amplifier is simple: it is the AC frequency component with the highest amplitude in the FFT (Fast Fourier Transform) of the signal. The lock-in will implement a very narrow pass-band filter around it, thoroughly reducing uncorrelated signals. However, the narrower the filter, the less definition the image has, as high-frequency components of the narrow band will also be reduced. After that, the final images are reconstructed using the inverse radon transform of the processed data.

**Figure 4:**
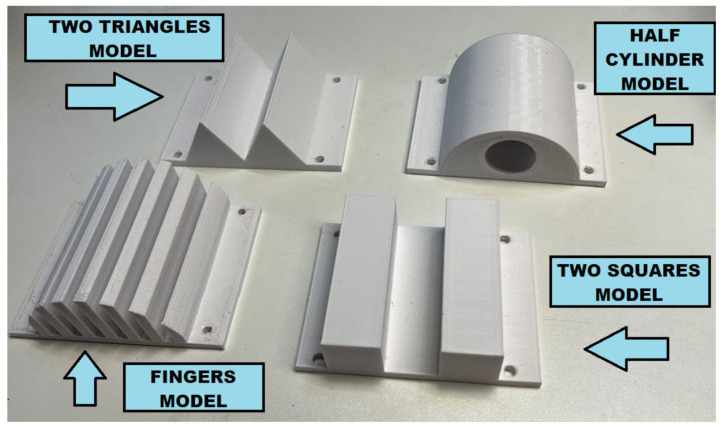
Models with different shapes and sizes are used as phantoms to evaluate the 3D Immersion Scanner’s ability to capture and reconstruct different types of objects.

Four 3D printed models were used, and their respective scans were performed: a Two Triangles model, the Half Cylinder model, the Fingers model, and the Two Squares model, as in Figure 4.

## 3. Results

**Figure 5:**
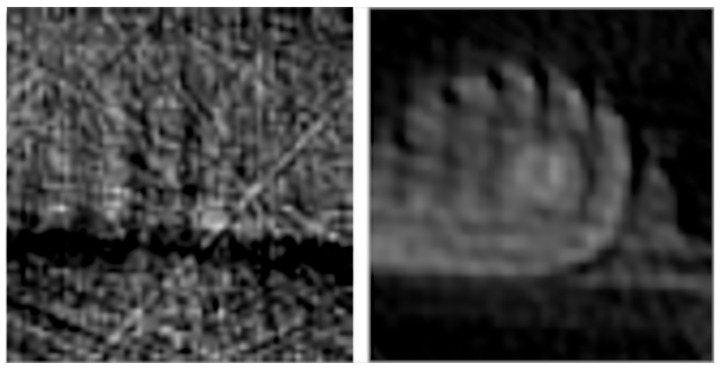
**Left**: Fingers model scanned using only water; **Right**: As damped step functions were now present in the signal due to the Xanthan gum, they could be used as an AC signal to the lock-in amplifier; the image is much clearer.

Our results demonstrate the reconstructed images from our 3D printed models, showing that this low-cost and relatively simple approach has the potential for further development and use in various applications. Figure 5 demonstrates the importance of the Xanthan gum in reducing the image’s noise.

**Figure 6:**
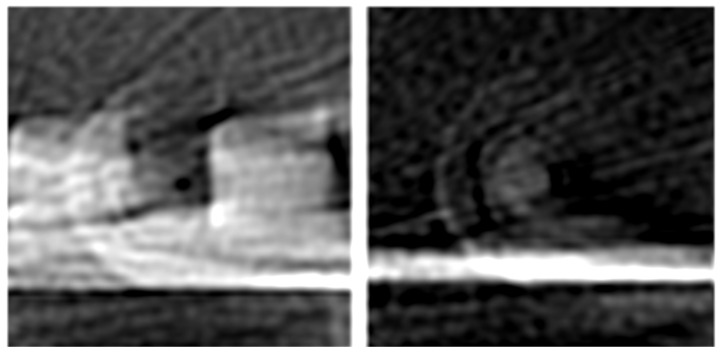
**Left**: The Two Squares model scan overlapped with an artifact; **Right**: This artifact can be more clearly seen when the platform is empty.

**Figure 7:**
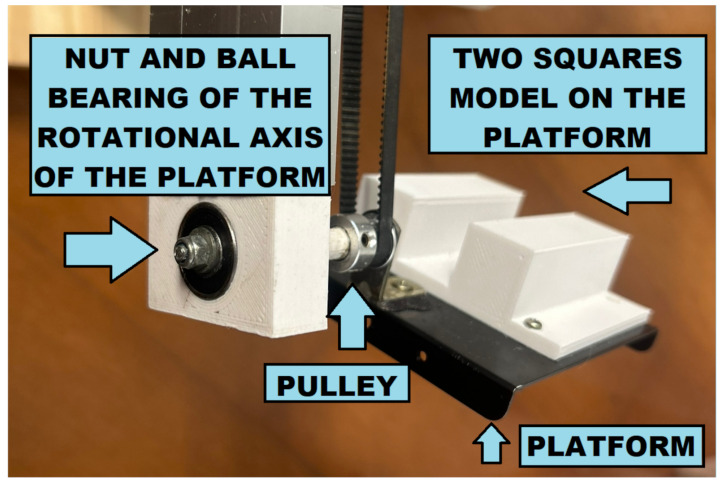
The mechanical setup of the platform (here with the two squares model) consists of a hexagonal nut, a ball bearing, and the pulley responsible for rotating the platform during scanning. However, as the rotational axis of the platform is close to the model’s center of mass, it overlaps and creates a watermark-like artifact.

Two major artifacts are present in the reconstructed images. The diagonal lines are due to the inverse radon transform having to interpolate data between every 3.6${}^{\circ }$. Reducing that angle provides images with higher resolution, but this also produces a proportional increase in scanning time. The second artifact is similar to a watermark, formed by round-shaped concentrical lines, one of which is hexagonal. This artifact can be seen when the platform is scanned without any model, as shown in the right part of Figure 6. These lines result from the overlap of the scanned images of the ball bearing, the pulley, and the hexagonal nut of the rotational axis of the platform, as shown in Figure 7.

The platform was constructed so that the center of mass of the platform plus the object is close to the rotation axis of the platform. This reduces the torque required to rotate the platform and minimizes vibrations, maintaining stability and accuracy. However, it also results in the appearance of a watermark-like artifact.

**Figure 8:**
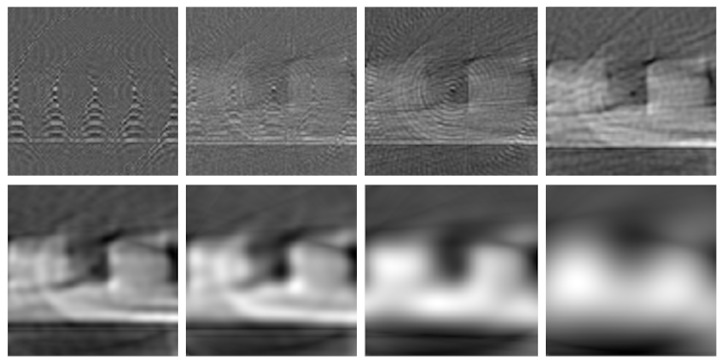
Different filter configurations showing different image reconstructions for the Two Squares model—from wider (**upper-left**) to narrower (**down-right**) pass band filter by changing the lock-in amplifier parameters. Narrower bands reduce the noise and the artifacts, but also the sharpness in the contour of the image.

Changing the band of the lock-in amplifier changes the SNR of the generated image, as in Figure 8. If the band is set too large, only noise can be seen. If too little, the scanned object is visible but more blurred. Reducing the band is like decreasing the high-frequency components of the scanned image, where noise and the artifacts described above are mostly present. However, by doing that, the image sharpness is also reduced.

The number of layers scanned should be large enough to provide the highest resolution for the reconstructed image, but this also means that small vibrations in the surface of the fluid previously in between layers may be amplified. Following the literature, we set the number of layers so that the margin of error of each layer does not overlap that of its neighbor layer. Using the *t*-test with 95% confidence measured for every one of the 100 layers at a single platform angle, there is an average noise of around 22.64 stepper motor steps. As the total amount of steps to vertically scan an object at a particular angle is around 6300 steps, 6300/22.64 ≈ 278 layers are the maximum possible resolution so that there is no overlap in the margin of error of each of the layers. Thus, a 100-layer scan seemed a reasonable compromise between scanning time, resolution of the reconstructed image, and noise. In this configuration, the total scanning time for one object was around 4.5 h.

If we increased the number of scanned layers, or decreased the angle between scans, the resolution would certainly increase. However, the time would increase as well. Several other factors influence the total scanning time, but it mainly follows the rule: the more time available, the higher the quality of the final imaging.

To evaluate if evaporation of the water in the tank and temperature drifts may also cause artifacts in the reconstructed image, the tank was left at room temperature for 24 h, at around 60% humidity, while its temperature was measured. Then, the evaporation and temperature drifts were calculated proportionally to the time of a regular 3D model scan.

Evaporation was responsible for a drift of around 428 steps per total scanning time. The temperature was responsible for a drift of around 25 steps per total scanning time. In comparison, the total scanning steps consist of 50 turns of 6300 steps each, giving an amount of 315,000 steps per total scanning time. Thus, evaporation and temperature are responsible for a drift of about 0.14%. Still, these drifts have long time constants, representing ultra-low frequency components in the spectrum of the acquired signal that are eliminated during the software lock-in amplifier.

**Figure 9:**
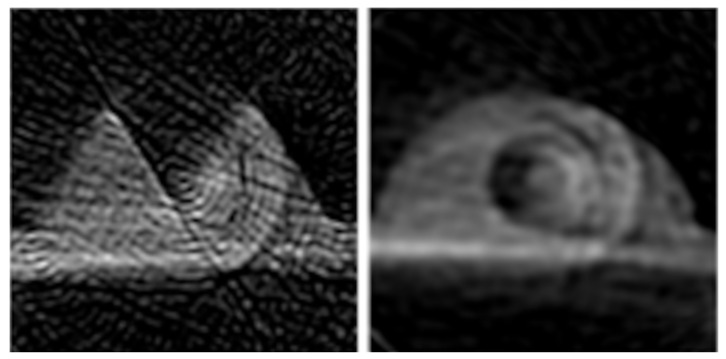
The 3D Immersion Scanner can reconstruct various model features, including flat, round, solid, and models with holes, such as the Half Cylinder and the Two Triangles models. **Left**: Two Triangles model scan; **Right**: Half Cylinder model scan.

Figure 9 demonstrates the image reconstruction using the Two Triangles and Half Cylinder models, displaying a range of configurations, including flat and curved lines.

To quantitatively evaluate the performance of the 3D Immersion Scanner, we compared the scanned Two Triangles model to its original CAD (Computer-Aided Design) model, which served as the basis for 3D printing this phantom.

**Figure 10:**
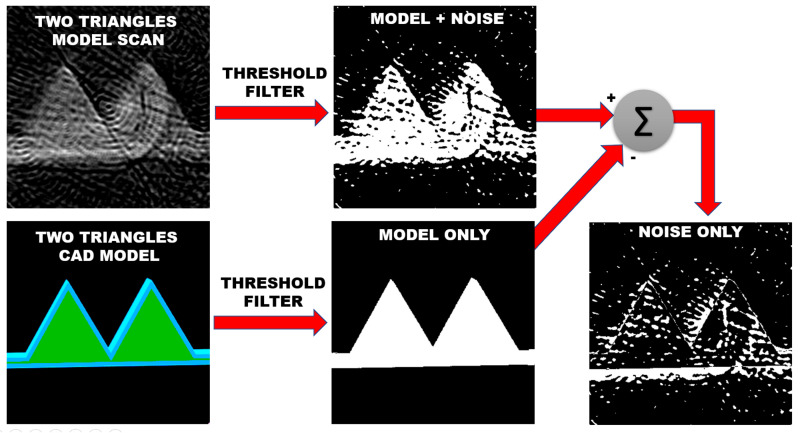
Two Triangles model scan (**Top**) and its original CAD model (**Bottom**) are compared to estimate the noise present in the 3D Immersion Scanning Technique.

The scan and the CAD images were passed through a threshold filter and converted into black-and-white versions; see Figure 10.

The threshold value was chosen so that 95% of the original power of the signal is still present in the images after the filter.

As the scan represents the CAD model and the experimental noise, an image representing the noise only was obtained as the absolute value of the difference between the filtered scan and filtered CAD models.

A ratio between the number of white pixels in the filtered CAD model (28,913 pixels) and the number of white pixels in this noise image (14,522 pixels) leads to an SNR of 20.log(28,913/14,552) = 5.9813 dB.

In addition, given that 14522 noise pixels are present in the total 352 × 352 pixels of the reconstructed image, they represent around 11.74% of the total amount of pixels of the reconstructed image.

**Figure 11:**
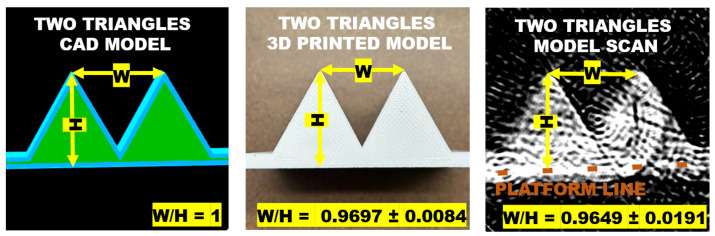
Width and height ratio of the Two Triangles model scan is compared to the same ratio of its 3D printed model.

The platform that holds the model during the scan can be seen as a white shade horizontal line underneath the reconstructed model; see the right part of Figure 10. The platform is also indicated by the dotted brown line of Figure 11. It leads to a few extra pixels computed as noise for a rough estimation of the SNR.

The 3D printed model has a width of 30.7200 ± 0.2388 mm and height of 31.6800 ± 0.3445 mm. Although the the ratio between the width (W) and height (H) of the Two Triangles CAD model is unitary, its 3D printed model has a ratio of 0.9697 ± 0.0084, calculated by using a *t*-test with 95% confidence. In comparison, the Two Triangles model scan has a ratio of 0.9649 ± 0.0191.

As the ratio of the scanned model overlaps that of the 3D printed model, the null hypothesis cannot be disproved, and the scan is likely to be consistent with the 3D model used in the scan.

The platform line again introduces imprecision in the height’s measurement in the scan image, so the height of the scan was measured from the top of the left triangle to the top of the platform line; see Figure 11.

## 4. Discussion

Making a 3D Immersion Scanner setup requires relatively little instrumentation compared to traditional CT scanners or 3D scanners in general.

Our technique relies on measuring the amount of water the submerged object displaces to reconstruct its 3D image. If the horizontal area of the tank is significantly larger than the area required to fit the object and the platform, the water level displacement caused by the submerging object will be small. As a result, the quality of the reconstructed image may be compromised due to the low signal-to-noise ratio. However, this does not mean the technique is limited to small objects. The approach can be scaled up to scan much larger objects as long as the horizontal area of the tank is close to that required for the object to fit in, a compromise between tank size and object size, to achieve the best possible image reconstruction quality.

In the scale of our experiment, the most significant difficulty was to have a precision of a few micrometers over a range of a few centimeters. Thus, we investigated different types of distance sensors.

Low-cost ultrasonic sensors, for instance, can measure a few centimeters but with errors of a few millimeters. They present echoes due to every single part of the object or mechanisms outside water. Low-cost LIDAR sensors have issues with the transparency of the water and not enough resolution [10,11,12,14,15,16].

The surface tension also plays an important role in choosing a level sensor for our application. Depending on the type of fluid, a sensor position with a certain distance to the reservoir walls needs to be chosen, as the meniscus can cause a mismatch between the liquid level and the sensor reading due to surface tension.

Electrodes tubes inserted vertically in the liquid measuring the fluid’s impedance, capacitive sensors outside the tank, or even optical measurements of the liquid in a transparent tank were also tried. Still, they all missed the measurements of a few layers.

A value of around 6 dB was calculated as the SNR of the 3D immersion scanner, but this depends on the specific parameters of the experiment, such as the sensor type, the filter coefficients, the size of the tank and the resolution of scanning.

The objects to be scanned should be impermeable to the fluid they are immersed in. Otherwise, the object may absorb some of the fluid, reducing the fluid level increment during the scan. In this case, dripping artifacts are also expected after the object is raised above the fluid, and partial fluid absorptions are expected in the next few layers, which may distort the reconstructed image.

As the fluid cannot penetrate the object’s material structure, the reconstructed image is limited to the object’s surface similar to 3D scanning technologies [1,4]. However, unlike traditional 3D scanning, which typically produces point clouds (set of data points in 3D space that represent the object’s surface) as the output, our method does not, as it generates a reconstructed 3D image of the object’s surface similar to the CT scan of the surface of an object.

Tomography produces a 3D representation from a series of 2D projections, typically carried out by taking X-rays of an object from different angles. Then, mathematical algorithms reconstruct a 3D image of the object. The same happens in our technique. However, instead of X-ray beams, we measure fluid level displacements acquired while submerging the object step-by-step at various angles.

Regarding the tank fluid, the Xanthan gum should be well mixed with water before the first experiment. An inhomogeneous mixture can lead to the formation of blobs, making the fluid level less precisely represent the submerged part of the object. Adding too much thickener can make the fluid too viscous, causing air bubbles to be trapped in the fluid every time the platform lowers or raises the object. When these bubbles pop, random artifacts can be expected.

Viscous liquids generally evaporate more slowly than less viscous ones because the molecules are more tightly packed together and require more energy to escape the surface and enter the vapor phase. Thus, the mixture of Xanthan gum and water reduces the evaporation of the fluid [17], making the water level drift less over time. Temperature is another aspect that influences the liquid level in the tank due to the volumetric dilation of the fluid. By connecting a water-cooled heat sink to an isothermal bath and submerging it into the tank, it took several hours for the fluid in the tank to get in equilibrium with the isothermal bath, demonstrating the high thermal inertia of the mixture. These scenarios show that the change in water level is a very slow phenomenon (at 22 ${}^{\circ }$C and 60% humidity). These variations behave as ultra-low frequency components in the spectrum of the acquired signal. The derivative naturally removes these signals in the data processing.

Corrosion of the needle could also generate long-term drifts in the measurements. Still, due to the low current provided by the 4.7 MΩ resistor, the electrode showed no need for replacement even after our set of experiments.

In terms of mechanics, the ball bearing used in the axis of the platform should have a level of precision so that the platform rotates but does not wobble around its axis. Otherwise, there are impacts on the precision of the reconstructed image. Using two co-axial ball bearings is usually a solution. However, as those ball bearings are mainly not water resistant, oxidation problems arise and they get stuck over time. They had to be replaced several times, even with a generous layer of water-repelling grease around them.

Unlike the linear slide stepper motor, the stepper motors used to move and rotate the platform are powered by high currents and generate a significant amount of electromagnetic noise that could potentially interfere with the comparator. The platform remains stationary while the needle takes fluid level measurements to mitigate this risk.

Our low-cost 3D scanning approach has achieved surface scanning of objects at an instrumentation cost of around 500 Euros, significantly less expensive than traditional 3D and CT scanners. Moreover, the costs of our scanning approach do not scale with the size of the object, as is the case for other scanners. In addition, very large objects can be scanned with our technique at relatively low instrumentation costs. However, given the early experimental stage of our technique, costs are still largely estimated.

Moreover, the cost to scan a single piece using our system is low and just limited to the electricity needed to move the motors, similar to that of a desktop 3D printer. Additionally, the cost of consumables such as water and Xanthan gum, which may need to be replaced after prolonged use, is negligible at just a few euros. In comparison, traditional scanners may require costly maintenance and repair over the lifetime of the equipment, which can significantly add to their overall cost.

### Future Works

Some of the limitations of the proposed method could be addressed in future works. While the reconstructed images generated by our prototype produced ultrasound-like scans of our 3D models, the image quality could be improved by several factors. Increasing the number of scanned layers and reducing the interpolation angle during the scan could provide more data for the reconstruction, resulting in higher-resolution images. For larger objects, which tend to displace more water, SNR can be increased, reducing the noise present. A higher SNR also requires wider-band filters, which can preserve the borders of the reconstructed images and increase their sharpness. An investigation on the relation between object size/tank surface size and SNR may also be part of future works.

Lowering the position of the platform’s pulley can prevent the generation of artifacts that overlap with the scanned object. Waiting for the vibrations in the fluid to decay can also reduce noise in the final image. However, these improvements come at the expense of longer scanning times or a more expensive setup, which could be implemented in future works. Future works could also address the platform’s presence in the reconstructed scan by removing it by post-processing or making it thin enough so that its presence may not be relevant in the reconstruction process.

To reduce scanning time, it may be possible to investigate the use of different fluids and their ability to dampen vibrations on their surfaces. Additionally, an array of level sensing sensors could be employed to acquire a more average fluid level along the surface, rather than waiting for the waves to dampen over time.

Object materials that may be permeable to water cannot be scanned with the current approach. However, such objects may be impermeable to oil or other organic fluids and thus such fluids might be used. Consequently, the materials of the object and the fluid need to be matched for such specific cases.

The use of ceramic ball bearings in the platform could also be explored to overcome the problem of oxidation with water-resistant ball bearings, despite the higher price.

As this paper aims at the proof of principle of the 3D Immersion Scanning Technique, objects symmetrically along their rotational axis were used for simplicity, but this does not stand when using the technique differently. Future research may address this issue by rotating and rescanning the object along multiple axes, using these data to fully reconstruct the object as a 3D model rather than an image scan, providing a more comprehensive representation of the object’s structure.

Similar to how the Radon transform can be viewed as a sum of the X-ray attenuations in a line of voxels in CT scanners, every 3D Immersion Scan is an overlap of multiple frames of the scanned object along its rotational axis. While it is possible to generate a complete 3D reconstruction by changing the object’s orientation and rescanning it in a controlled manner, this would require improvements in the degree of freedom of the platform. This approach would increase the scanning time, and this should also be addressed in future works.

Regarding scalability costs, our technique can easily be adapted to larger object sizes by using a larger tank and larger linear slides, with costs that would grow approximately proportionally to the size of the parts. Therefore, our low-cost 3D scanning approach has significant potential for cost savings in various industries and applications.

Despite these potential improvements, our technique shows promise as a relatively simple and cost-effective approach to producing images of various objects, with several advantages over traditional 3D and CT scanners.

## 5. Conclusions

The 3D Immersion Scanning Technique offers a low-cost and versatile alternative to traditional CT and optical scanners. As no light is involved in scanning the object, this technique can scan objects of various types and shapes, including those that are reflective, transparent, or have gaps and irregularly shaped openings.

In this technique, CT scan technology is used to reconstruct the 3D image of an object without the need for dangerous X-rays. This is achieved by measuring the fluid level displacement during the submersion of the object, using a needle tapping the surface of the fluid.

While there are limitations to this technique, such as low resolution and a long-scanning time, it can easily be scaled up for larger objects by using larger tanks. Moreover, there is an optimal choice between tank size and object size to achieve the best possible image reconstruction quality.

The main challenge in implementing this technique is achieving a high level of precision with low noise in measuring the fluid level displacement over a range of several centimeters. This requires careful consideration in choosing the appropriate fluid level sensor and fluid type.

Overall, the 3D Immersion Scanning Technique can contribute to the field of 3D scanning and imaging, particularly for industries and applications that may not have the resources to invest in expensive CT or 3D scanners.

As further research is conducted and improvements are made to this technique, the 3D Immersion Scanning Technique has the potential to be an accessible and practical solution for various fields and applications.

## Data Availability

The data are available from the first author upon reasonable request.

## Abbreviations

The following abbreviations are used in this manuscript:

3D	Three Dimensional
CT	Computer Tomography
LIDAR	Light Detection and Ranging
SNR	Signal-to-Noise Ratio
MATLAB	Mathematical Programming Language
T-Test	Statistical Mean Comparison Test
CAD	Computer-Aided Design

## References

[1] Marcy A., Fruciano C., Phillips M., Mardon K., Weisbecker V. (2018). Low resolution scans can provide a sufficiently accurate, cost-and time-effective alternative to high resolution scans for 3D shape analyses. PeerJ.

[2] Rowe A., Rayfield E. (2022). The efficacy of computed tomography scanning versus surface scanning in 3D finite element analysis. PeerJ.

[3] Verykokou S., Ioannidis C. (2023). An Overview on Image-Based and Scanner-Based 3D Modeling Technologies. Sensors.

[4] Haleem A., Javaid M. (2019). 3D scanning applications in medical field: A literature-based review. Clin. Epidemiol. Glob. Health.

[5] Mendricky R., Sobotka J. (2020). Others Accuracy Comparison of the Optical 3D Scanner and CT Scanner. Manuf. Technol..

[6] Pleszczyński M. (2021). Implementation of the computer tomography parallel algorithms with the incomplete set of data. PeerJ Comput. Sci..

[7] Souza M., Alka Cordeiro D., Oliveira J., Oliveira M., Bonafini B. (2023). 3D Multi-Modality Medical Imaging: Combining Anatomical and Infrared Thermal Images for 3D Reconstruction. Sensors.

[8] Fathy G., Hassan H., Sheta W., Omara F., Nabil E. (2021). A novel no-sensors 3D model reconstruction from monocular video frames for a dynamic environment. PeerJ Comput. Sci..

[9] Göldner D., Karakostis F., Falcucci A. (2022). Practical and technical aspects for the 3D scanning of lithic artefacts using micro-computed tomography techniques and laser light scanners for subsequent geometric morphometric analysis. Introducing the StyroStone protocol. PLoS ONE.

[10] Jafri S., Shamim S., Faraz S., Ahmed A., Yasir S., Iqbal J. (2022). Characterization and calibration of multiple 2D laser scanners. PLoS ONE.

[11] Castillón M., Palomer A., Forest J., Ridao P. (2019). State of the art of underwater active optical 3D scanners. Sensors.

[12] Palomer A., Ridao P., Youakim D., Ribas D., Forest J., Petillot Y. (2018). 3D laser scanner for underwater manipulation. Sensors.

[13] Gonçalves R., Haueisen J., Marques J. (2020). Inductive temperature measurement: A new sensor improvement for industrial applications. Rev. Sci..

[14] Himri K., Ridao P., Gracias N. (2021). Underwater object recognition using point-features, bayesian estimation and semantic information. Sensors.

[15] Lou L., Li Y., Zhang Q., Wei H. (2023). SLAM and 3D Semantic Reconstruction Based on the Fusion of Lidar and Monocular Vision. Sensors.

[16] González-Merino R., Sánchez-López E., Romero P., Rodero J., Hidalgo-Fernández R. (2021). Low-Cost Prototype to Automate the 3D Digitization of Pieces: An Application Example and Comparison. Sensors.

[17] Berninger T., Dietz N., Gonzalez Lopez O. (2021). Water-soluble polymers in agriculture: Xanthan gum as eco-friendly alternative to synthetics. Microb. Biotechnol..

